# Acute malnutrition associated with mid-upper arm circumference among under-five children in tribal areas, India: a cross-sectional study

**DOI:** 10.1017/S1368980024002465

**Published:** 2024-12-05

**Authors:** Shraboni Patra, Shashikant Sambharkar, Sheetal Harode, Kalpana Barde, Amita Pillewan

**Affiliations:** 1 State Monitoring and Resource Cell, Nutrition Bureau, Nagpur, MH, India; 2 Public Health Department, Nutrition Bureau, Nagpur, MH, India; 3 Nutrition Bureau, Nagpur, MH, India

**Keywords:** mid-upper arm circumference, weight-for-height z scores, moderate acute malnutrition, severe acute malnutrition, under-five tribe children

## Abstract

**Objective:**

For the past three decades, India has implemented several nutrition programmes to address malnutrition in the under-fives. To understand the programme’s impact, this study assesses the prevalence of acute malnutrition, moderate acute malnutrition (MAM) and severe acute malnutrition (SAM), using mid-upper arm circumference (MUAC) among tribal children.

**Design:**

The survey was conducted in two tribal blocks (Desaiganj and Bhamragad) of the Gadchiroli district in Maharashtra to identify children registered in the ‘Anganwadi’ program.

**Setting:**

A community-based cross-sectional survey was carried out.

**Participants:**

The total sample size was 1055 children (aged 0–59 months).

**Results:**

The overall prevalence of SAM and MAM was 1·4 % (*n* 15) and 9·8 % (*n* 103). A higher prevalence of MAM was found in males (38·5 %, *n* 40) and females (27·1 %, *n* 28) in below 6 months. Additionally, a higher prevalence of MAM was observed in females (10·7 %, *n* 113) compared with males (9·0 %, *n* 95). The prevalence of SAM was significantly (*P* < 0·001) higher in females (1·7 %, *n* 18) than in males (1·0 %, *n* 11). Children aged between 12 and 17 months were sixteen times more likely (OR = 16·9, *P* < 0·001, CI = 4·8, 59·6) to have MAM (MUAC < 12·5 cm) than children aged between 6 and 11 months. Children from the Desaiganj block were significantly less likely (OR = 0·4, *P* < 0·001, CI = 0·2, 0·7) to have MAM compared with children from Bhamragad. Approximately 4 % (*n* 42) of children were classified as critically malnourished.

**Conclusion:**

There is an urgent need for block-level monitoring of MAM and SAM, as well as evaluation of existing nutrition programmes, to address the disparity in the sex-specific prevalence of MAM and SAM in tribal areas.

Child malnutrition is a persistent issue and a major contributor to disease burden in India^(1)^. An estimated 7·3 % (50 million) of all children under five suffer from wasting at any given time^(2)^. Wasting (low weight for height) is one of the basic indicators for assessing the severity of the health status of children. Acute malnutrition is a form of undernutrition caused by decreased food consumption and illness that results in sudden weight loss^(3)^. The Joint Child Malnutrition Estimates reveal insufficient progress to reach the 2025 World Health Assembly global nutrition targets and Sustainable Development Goal target 2·2. An assessment of progress towards the wasting target is not possible for nearly half of countries, and a very high prevalence of under-five child wasting is considered a serious threat to child mortality in India^(4)^.

According to new data, child malnutrition might be worsening. Though fewer children in India are dying, and those who survive are more malnourished and anaemic in many states^(5)^. The crisis of child malnutrition in India has often been attributed to historical antecedents such as poverty, inequality and food shortage. However, countries with similar historical and societal makeup and comparable per capita income have fared much better. Further, the immediate impacts of COVID-19 left several children acutely malnourished, as key health, nutrition and other life-saving services became less accessible^(3)^.

An easy, accurate and low-cost indicator is helpful for early identification of children with acute malnutrition. Therefore, weight-for-height z scores (WHZ)^(3,6)^ and mid-upper arm circumference or MUAC (as subcutaneous fat and muscle mass decrease in undernourished children) have been used to screen for acute malnutrition^(7,8)^. ‘Acute malnutrition’^(9)^ can be divided into moderate acute malnutrition (MAM) with a WHZ between –2 and –3 Z-scores (sd) or a MUAC between 115 mm and 125 mm., and severe acute malnutrition (SAM), being defined as a WHZ < –3 sd or a MUAC of < 115 mm^(10,11)^. WHZ has been used for years in clinical settings for diagnosing SAM^(12)^. The use of MUAC was introduced with the development of community-based management of SAM^(13)^. The WHO standards for MUAC for age show that in a well-nourished population, there are very few children aged 6–69 months with a MUAC of < 115 mm, but in an underprivileged population, the percentage of SAM (with a MUAC < 115 mm) in children aged 6–59 months climb up^(10)^. Though MUAC or Weight-for-age Z score (WAZ) cut-offs vary a lot in different populations, even within the countries a study^(14–16)^ shows that the combined case definition (WAZ less than –3 sd and MUAC < 115 mm) is found more effective in detecting the severity of malnutrition and preventing child mortality than considering WAZ less than –3 sd or MUAC < 115 mm alone. MUAC is closely related to the risk of dying and is easy to implement at the community level after minimum training by health workers or even by volunteers^(17)^. MUAC and WHZ, however, do not identify the same set of children as having malnutrition and using only one of the diagnostic criteria proposed by WHO may potentially leave some high-risk children untreated^(18)^. As they are additive and not complementary, it would be pragmatic to retain both criteria for admission to treatment programmes^(14)^.

Though the prevalence of undernutrition (i.e. stunting, wasting and underweight) among tribal children under 5 years in India has reduced from 43·8 %, 27·4 % and 45·3 % in National Family Health Survey (NFHS)-4 to 40·9 %, 23·2 % and 39·5 % in NFHS-5, respectively, the prevalence of malnutrition in under-five children is a grievous concern.^(19,20)^. As per the 2011 Census, 10·5 million tribal people are living in Maharashtra and Gadchiroli district has a 38·71 % Scheduled Tribe population which is the highest in the Nagpur division^(21)^. Within Maharashtra, all parameters of undernutrition, i.e. stunting (41·4 %), wasting (32 %) and underweight (46·5 %), even in their severity (18·3 %, 13·5 % and 20·2 %, respectively), are highest among the tribal children^(19,20)^.

Tribal people are the most underprivileged in terms of their hardship to thrive for better education and skill enhancement and the adversity they experience in income opportunities available and accessible to them, which hinders their overall development. Uncertain modes of employment and economic instability lead to poverty, which makes them victims of morbidity and malnutrition^(21,22)^. There is wide acknowledgement that excess morbidity and poor nutritional status in childhood for tribes are partly due to poverty and food insecurity and partly due to poor access to healthcare services^(22)^. The nutritional status of the children of the Indigenous tribe, in India as well as in the world, has not received much attention, and there is a lack of focused surveys to obtain data on their food habits and nutritional status. Hence, there is a large scope to explore the nutritional status and the underlying causes of malnutrition among tribal children. The present study contributes to understanding the status of undernutrition among under-five children belonging to the indigenous tribal population in the Indian context.

The ‘APJ Abdul Kalam Amrut Yojana’ was launched in 2015 by the ‘Tribal Development Department’ of the Government of Maharashtra aims to provide at least one nutritious meal to pregnant and lactating women at the ‘Anganwadi Centre’ (courtyard shelter). Under the scheme, every tribal woman in her third trimester of pregnancy is entitled to a free nutritious hot meal daily continuing for 3 months post-delivery so that the newborn is healthy. However, to the best of our knowledge, there has not been a single study to check the nutritional scenario, whether it has improved or not in the tribal blocks of Gadchiroli district. Against this backdrop, the present study has been undertaken with a specific objective to assess the current scenario of undernutrition among children below 5 years of age in Desaiganj (formerly known as Wadasa) and Bhamragad blocks, which are predominantly a tribal area in Gadchiroli district^(21)^. Hence, the objective of the present study is to assess the current prevalence of acute malnutrition in terms of MAM and SAM using MUAC among children aged 0–5 years in the Gadchiroli district of Maharashtra.

## Methods

### Study area

Gadchiroli district, located in the north-eastern side of Maharashtra, shares borders with Telangana and Chhattisgarh. The district is classified as tribal and underdeveloped with most of the land covered by forest and hills, accounting for about 76 % of its geographical area. The population of Scheduled Castes and Scheduled Tribes in the district is 11·25 % and 38·7 % respectively^(21)^. The survey was conducted in two purposively selected tribal blocks, Desaiganj and Bhamragad blocks in the Gadchiroli district.

### Study design and sampling

A community-based cross-sectional survey was conducted in six districts of Vidarbha region, Maharashtra by the Nutrition Bureau, Public Health Department, between December 2021 and March 2022.

**Fig. 1:**
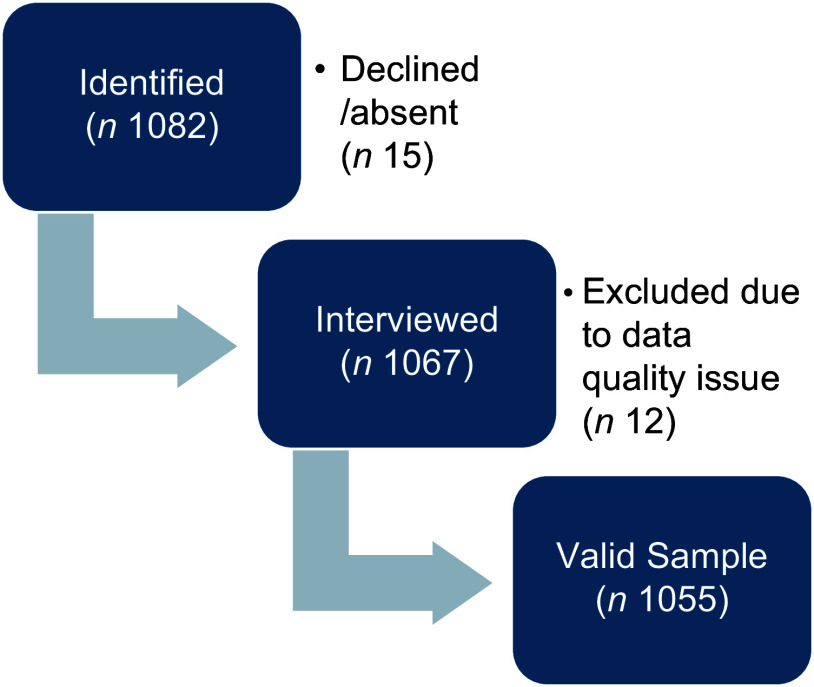
Flowchart showing sample exclusion.

The aim was to assess the nutritional status and dietary intake of children under five and pregnant women and lactating mothers registered with the Integrated Child Development Services^1^ through Anganwadi centres. Detailed information on pregnant women, lactating mothers (within 1 year of delivery) and children (0–5 years) was collected from the respective Anganwadi centres through house-to-house visits. The present study focuses on the analysis of children’s anthropometric measurements from this nutrition survey. A stratified simple random sampling method was used in the study. Gadchiroli, being a rural district with a 38·7 % tribal population, was divided into two categories based on the 2011 Primary Census: urban population and 100 % rural population. Blocks with 100 % rural population were further categorised based on the number of tribal populations. From each group, two blocks were randomly selected: Desaiganj (< 5000 Scheduled Tribe households) and Bhamragad (> 5000 Scheduled Tribe households). Inhabited villages in the selected blocks were grouped by population size (below 500, 500–1000 and above 1000) and randomly selected for inclusion in the survey. A total of twenty-six villages were covered, with 1055 children under 5 years of age registered at the Anganwadi centres included in the study. See Fig. 1 for the sample exclusion criteria.

### Sample

**Fig. 2:**
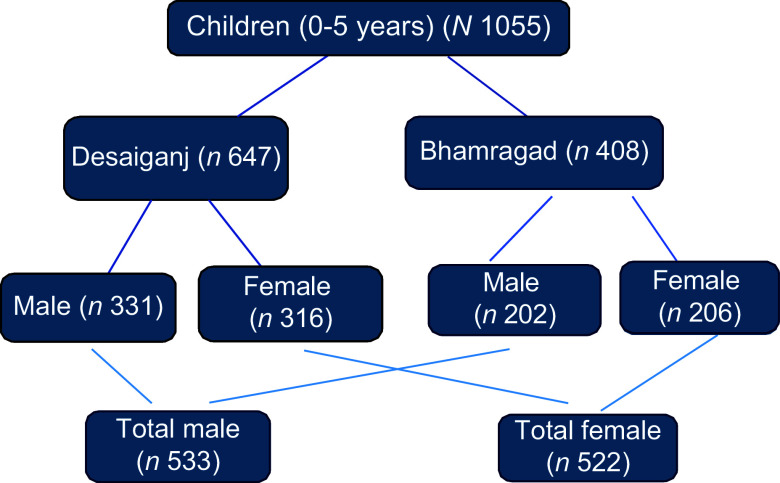
Sample distribution.

The total sample considered for analysis was 1055 children (0–59 months) from the Anganwadi centres of the selected villages in the survey period (Fig. 2).

### Anthropometric investigation

The procedure followed in taking anthropometric measurements was as per the United Nations and using the WHO child growth charts/standards. The Z score values (WAZ, Height-for-age Z Score (HAZ) and WHZ) were classified by the WHO^(6)^. Anthropometric measurements such as height and weight of mothers and their children (0–5 years) in addition to MUAC were measured using standardised equipment and procedures, i.e. MUAC were measured using the WHO method with two independent observers’ methods, with both repeating if the measures were not within a pre-specified limit of agreement.

Height/length Measurement: The length of the children was measured using an ‘infantometer*’,* with a headboard and sliding foot piece, placed on a stable level. Diapers, shoes, socks, braids and hair ornaments were removed. The length/height of the children under 2 years was measured by lying them and for the older children measured by standing. During measurement, hands were kept on the shoulder/knees and pressed gently without hurting the child. The soles of the feet were flat on the foot piece pointing up. Measurements were taken to the nearest 0·1 cm.

A stadiometer was used to measure standing height with a vertical backboard, a fixed baseboard and a movable headboard placed on a level floor. During measurement, socks, shoes, hair ornaments and braids were removed. One person kneeled near the child’s foot and helped the child to stand with the back of the head, shoulder blades, buttocks, calves and heels touching the vertical boards. At the same time with the other hand, the headboard was pulled firmly on the top of the head to compress hair. Measurements were taken to the nearest 0·1 cm.

Weight Measurement: The weighing scale, used to measure the child’s weight, was a ‘*Spring Balance’,* which was solidly built, durable, electronic (Digital), able to measure with a precision of 0·01 kg (10 g) and allowed taring (the act of zeroing the instrument). Hence, there was no need to subtract weight, reducing the risk of error. First, the child kept calm by holding him/her in the mother’s arms. A beam scale or a hanging scale (Salter type) was used where a tared machine was not available. Before taking weight, the scale was adjusted to zero. Time was given for the child to settle and the weight to stabilise.

MUAC measurement was done with a tri-coloured tape known as Shakir’s tape (Annexure 1·1). MUAC was taken for children aged 6–59 months only. The arm circumference was measured on the upper left arm by flexing the child’s elbow to 90 degrees. Then, the midpoint between the acromion process of the shoulder joint and the tip (olecranon) of the elbow was marked and then the arm was allowed to hang freely. At the same time, the measuring tape was placed snugly around the arm at the midpoint mark. The tape was not pulled too tightly during the measurement.

### Data collection

Data were collected using a pretested semi-structured interview schedule through personal interviews. A single interviewer collected the data by conducting face-to-face interviews with the mothers of eligible children. All those who fulfilled the inclusion criteria were eligible to participate in the study.

### Variables

Independent variables include the *child’s age* (categories below 6, 6–11, 12–17, 18–23, 24–35, 36–47 and 48–59 months), *child’s sex* (male and female), *blocks* (Bhamragad and Desaiganj) and *wasting* (WHZ less than –2 sd, yes and no) used in logistic regression analysis.

Dependent variables include moderate acute malnutrition or MAM (MUAC < 12·5 cm, yes and no), *severe acute malnutrition* or SAM (MUAC < 11·5 cm, yes and no) and severe wasting (WHZ less than –3 sd, yes and no).

### Statistical analyses

Simple bivariate and multivariate analyses were carried out to analyse the data. Bivariate logistic regression analysis was done to assess the effect of independent variables (age, sex, block and wasting) on the prevalence of MAM. The significance level of the association between the variables was shown by Pearson’s correlation coefficient and *χ* ^2^ values.

The Z score value (i.e. sd) for the anthropometric measures in terms of height for age, weight for age and height for weight was calculated using the WHO Anthro, version 3.2.2. Computation of all other variables and all statistical analyses were carried out by IBMSPSS Statistics, version 29.

### Findings

#### Characteristics of the study population

Table 1A represents the characteristics of the sample (0–59 months). Approximately 10 % (*n* 103) of children are under 6 months old, while about 33 % (*n* 347) are between 6 and 24 months old. The mean age of the sample is 28·6 months. The percentage of male children (50·5 %) is almost equal to the percentage of female children (49·5 %). Around 39 %, (*n* 408) of the sample is from the Bhamragad block, while the majority (61 %, *n* 647) is from the Desaiganj block. The mean MUAC, height and weight of the sample are 13·6 cm, 80·9 cm and 9·9 kg, respectively. The median age is 28 months.

**Table 1A.** Characteristics of children (0–59 months), Gadchiroli, Maharashtra, India, 2022

Characteristics	Percentage	Children (*n*)
Age group (months)		
< 6	9·8	103
6–11	11·2	118
12–17	12·5	132
18–23	9·2	97
24–35	19	200
36–47	19·4	205
48–59	19	200
Sex		
Male	50·5	533
Female	49·5	522
Block		
Bhamragad	38·7	408
Desaiganj	61·3	647
Total	100	1055
Mean age (months)		28·6
sd		17·04
Median		28
IQR		30
Mean MUAC (cm)		13·6
sd		1·18
Mean height (cm)		80·9
sd		12·62
Mean weight (kg)		9·9
sd		2·78

IQR, interquartile range.

The proportion of children with SAM is 1·4 % (*n* 15), whereas the proportion of children with MAM is 9·8 % (*n* 103) (Table 1B).

**Table 1B.** Nutritional status of children (0–59 months) Gadchiroli, Maharashtra, India

Malnutrition type	Percentage	Children
SAM	1·4	15
MAM	9·8	103
Normal	88·8	928
Total	100	1055

MAM, moderate acute malnutrition; SAM, severe acute malnutrition.

#### Prevalence of malnutrition among children between 0 and 59 months

Table 2 shows the prevalence of MAM and SAM among children aged 0–59 months based on their age group and sex in the selected blocks of the Gadchiroli district.

**Table 2.** Percentage of children (0–59 months) with moderate acute malnutrition (MAM, WHZ < 2 sd) and severe acute malnutrition (SAM < 3 sd) by age and sex in Gadchiroli, Maharashtra, India, 2022

Age group (months)	MAM (%)			SAM (%)			Acute malnutrition = MAM + SAM	*χ* ^*2*^ value Sex	Children (*n*)
	Male	Female	Total	Male	Female	Total			
								150·302, *P* < 0·001	
< 6	38·5	27·1	33·0	3·8	12·5	8·0	41·0		103
6–11	17·2	11·9	14·5	3·4	1·7	2·6	17·1		118
12 to17	8·6	16·4	12·2	1·4	1·6	1·5	13·7		132
18–23	12·2	22·9	17·5	0	0	0	17·5		97
24–35	4·3	11·3	8·0	0	0	0	8·0		200
36–47	0·9	2·1	1·5	0	0	0	1·5		205
48–59	1·0	0	0·5	1·0	1·0	1·0	0·5		200
Total	9·0	10·7	9·8	1·0	1·7	1·4	11·2		1055

MAM, moderate acute malnutrition; SAM, severe acute malnutrition; WHZ, weight-for-height z scores.

The results reveal that the malnutrition prevalence is 11·2 % in the study population, with MAM at 9·8 % and SAM at 1·4 %. MAM is the highest (33·0 %) in children under 6 months and decreases with age. The lowest MAM prevalence is in the age group above 47 months. Sex- and age-specific differences in MAM prevalence are noted with significant differences across all age groups. Higher MAM prevalence is found in males (38·5 %) and females (27·1 %) in the youngest age groups. Overall, girls (12·3 %) have a higher MAM prevalence than boys (10·1 %). Severe acute malnutrition (SAM) prevalence is 1·4 %, higher in females (1·7 %) than males (1·0 %). Children under 6 months show the highest SAM prevalence (8 %), decreasing with age group. The overall acute malnutrition (MAM + SAM) is 11·2 %, the highest in children under 6 months (41 %). A significant difference (*P* < 0·001) in MAM and SAM prevalence by sex is observed.

**Fig. 3:**
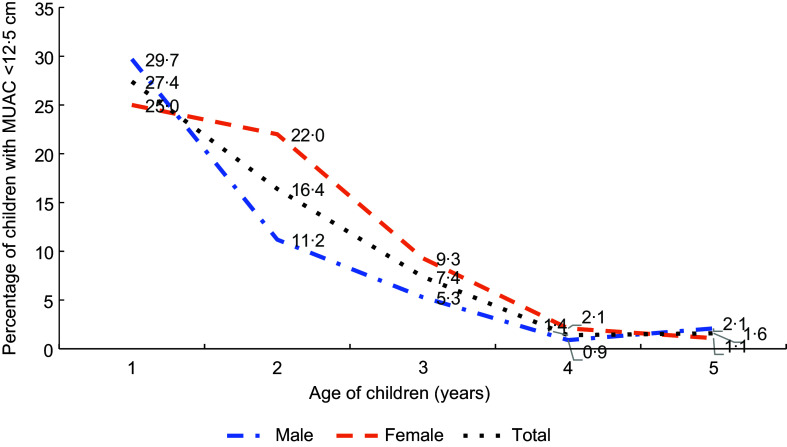
Prevalence of acute malnutrition (MUAC < 12·5 cm) in children 0–59 months, Gadchiroli, Maharashtra, India, 2022.

Figure 3 illustrates age- and sex-specific MAM prevalence among children aged 0–5 years. While MAM prevalence decreases with age, girls show higher moderate malnutrition rates than boys in subsequent age groups.

#### Prevalence of moderate acute malnutrition and severe acute malnutrition in selected blocks

**Fig. 4:**
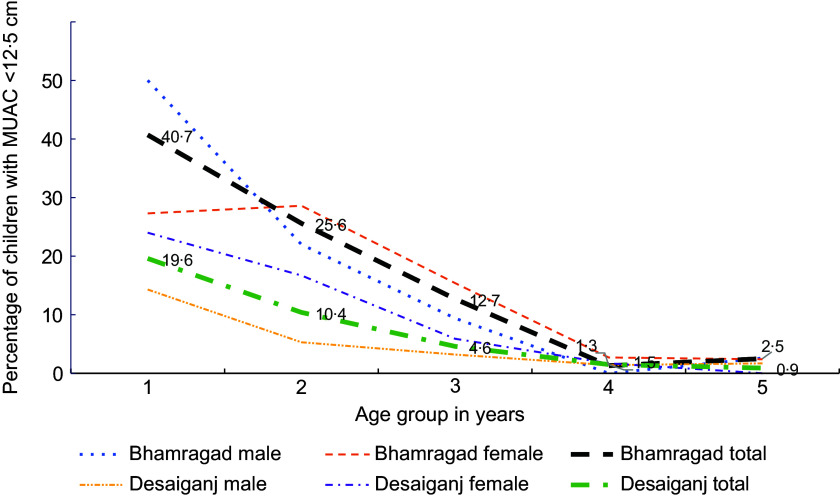
Prevalence of acute malnutrition (MUAC < 12·5 cm) in children 0–59 months, Gadchiroli, Maharashtra, India, 2022.

Figure 4 shows MAM prevalence among children aged 0–5 years with MUAC < 12·5 cm in selected blocks (Bhamragad and Desaiganj) in Gadchiroli district. MAM prevalence decreases with age in Bhamragad compared with Desaiganj. Both blocks have the highest prevalence below 1 year (40·7 % in Bhamragad, 19·6 % in Desaiganj) and lowest above 4 years (2·5 % in Bhamragad, 0·9 % in Desaiganj). Girls have higher MAM prevalence, especially in the age group of 2–4 years.

The figure shows that the prevalence of MAM has steadily decreased with the increase in the age group of the children in Bhamragad compared with the prevalence of MAM in children of Desaiganj block. Both blocks have observed the highest prevalence in the age group below 1 year (40·7 % and 19·6 % in Bhamragad and Desaiganj, respectively) and lowest in the children aged above 4 years (2·5 % and 0·9 %). In both blocks, girls are showing a higher prevalence of MAM particularly in children aged between 2–4 years.

#### Factors associated with moderate acute malnutrition among children (6–59 months)

Table 3 represents the result from bivariate logistic regression analysis in terms of the effect of age, sex, block and wasted (WHZ less than –2 sd) status of children 6–59 months on the prevalence of moderate acute malnutrition or MAM (MUAC < 12·5 cm). Results show that there is a significant decrease in the prevalence of MAM in the higher age group. Children in the age group 12–17 months are sixteen times more likely (OR = 16·885, *P* < 0·001) to have MAM (MUAC < 12·5 cm) than the reference category children (i.e. children aged 6–11 months).

**Table 3.** Bivariate logistic regression analysis showing prevalence of MAM among children (6–59 months) by their background characteristics, Gadchiroli, Maharashtra, India, 2022

Characteristics	MAM (MUAC < 12·5 cm) in percentage	OR (Exp-*β*)	*P*-values	95 % C.I. for Exp (*β*)
				Lower, Upper
Age group (months)				
6–11®	16·9	1		
12–17	13·6	16·9	< 0·001	4·8, 59·6
18–23	17·5	11·4	< 0·001	3·2, 40·7
24–35	8·0	15·3	< 0·001	4·2, 54·7
36–47	1·5	7·3	< 0·01	2·0, 25·9
48–59	1·5	1·1	0·243	0·2, 5·3
Child’s sex				
Male®	6·7	1		
Female	9·5	1·4	0·211	0·9, 2·4
Blocks				
Bhamragad®	12·0	1		
Desaiganj	4·9	0·4	< 0·001	0·2, 0·7
Wasting (WHZ < –2 sd)				
No®	5·3	1		
Yes	18·5	3·5	< 0·001	2·1, 5·9
Total	8·1			

MAM, moderate acute malnutrition; WHZ, weight-for-height z scores.

Reference category is ®.

Female children (OR = 1·45) are more likely to have MAM than male children. Children from the Desaiganj block (OR = 0·423, *P* < 0·001) are significantly less likely to have MAM than the children from the Bhamragad block. Children who are moderately wasted (WHZ less than –2 sd) are three times (OR = 3·510, *P* < 0·001) more likely to have MUAC < 12·5 cm than the children who are not wasted.

#### Prevalence of moderate acute malnutrition and severe acute malnutrition concerning mid-upper arm circumference and weight-for-height z scores in children (6–59 months)

**Fig. 5:**
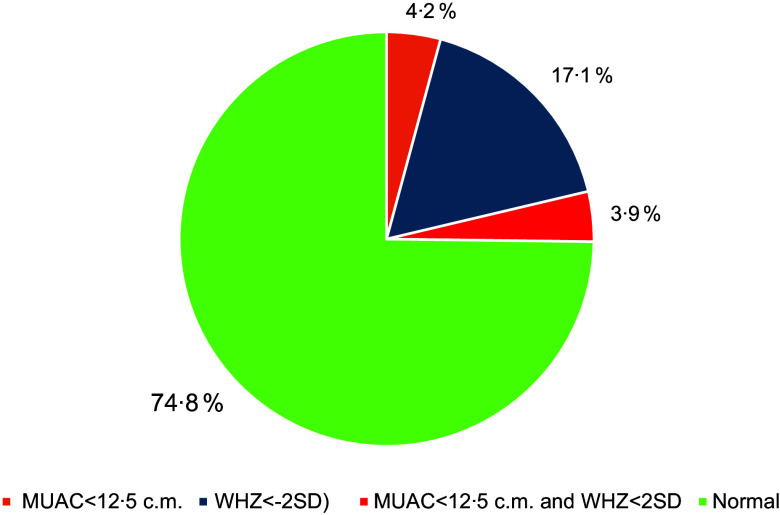
Percentage of children of 6–59 months with MAM (MUAC < 12·5 cm and WHZ < –2 sd), Gadchiroli, Maharashtra, India, 2022. WHZ, weight-for-height z scores. MAM, moderate acute malnutrition; WHZ, weight-for-height z scores.

Figure 5 represents the percentage of children aged between 6 and 59 months who have MAM in terms of having both MUAC < 12·5 cm and WHZ less than –2 sd. About 17 % and 4·2 % of children are found to be moderately malnourished for MUAC (< 12·5 cm) and wasting (WHZ < 2 sd). It is also found that about 3·9 % of children are critically malnourished as having both MUAC < 12·5 cm and WHZ < 2 sd.

**Fig. 6:**
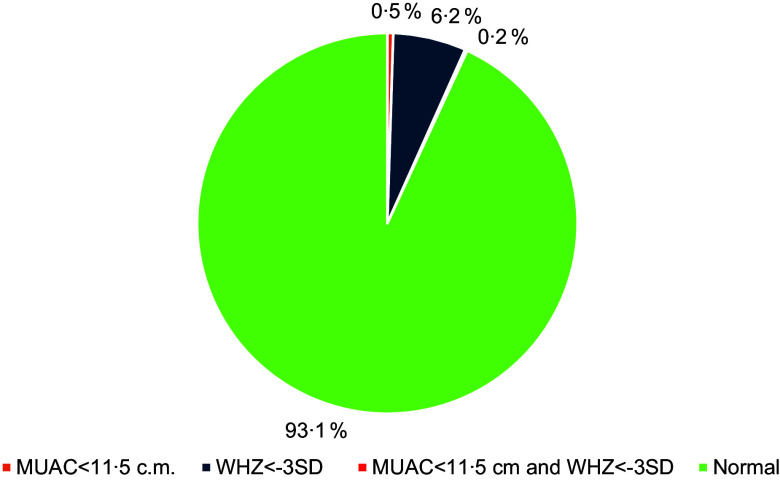
Percentage of children of 6–59 months with SAM (MUAC < 11·5 cm and WHZ less than –3 sd), Gadchiroli, Maharashtra, India, 2022. SAM, severe acute malnutrition; WHZ, weight-for-height z scores.

Figure 6 represents the percentage of children who are severely acutely malnourished (SAM) for both parameters, i.e. MUAC < 11·5 cm and WHZ < 3 sd. About 6·2 percent (WHZ < –3 sd) and 0·5 per cent (MUAC < 11·5 cm) of children are found to be severely acutely malnourished. The percentage of children having both WHZ less than –3 sd and MUAC < 11·5 cm is 0·2 per cent, who are considered critically ill and need special treatment.

## Discussion

The study found that acute malnutrition was more prevalent among female children aged 0–5 years compared with male children. Disparities in child malnutrition prevalence were observed at the block level, even with similar nutrition programs in place. Children in the Bhamragad block had a higher prevalence of acute malnutrition compared with those in the Desaiganj block with female children more affected than male children^(23)^. Regression analysis indicated significant differences in malnutrition prevalence based on age and sex^(24)^ in tribal blocks. A higher percentage of children with MUAC < 12·5 cm was wasted compared with non-wasted children. The present research observed about 4 % of children in the study area with both WHZ < 2 sd and MUAC < 12·5 cm who need to be considered critically ill. Further, about 7 % of children who are severely malnourished have a higher risk of morbidity and mortality^(22)^. The study highlighted the higher prevalence of acute malnutrition in children under 2 years^(7,25)^.

The prevalence of acute malnutrition among children under 5 years was 11·2 %, with 1·4 % experiencing SAM. Children with SAM are at significantly higher risk of morbidity and mortality. The study emphasised the need for urgent policy attention in districts like Gadchiroli to address the higher prevalence of SAM. Despite some improvements, child malnutrition rates in Maharashtra remained largely unchanged with a higher prevalence of wasting among children under 2 years in Gadchiroli compared with the state average. According to NFHS-5, the percentage of children (below 2 years) who are underweight (36 %) or wasted (26 %) has not changed since NFHS-4^(19,20)^. Though the prevalence of stunting (35·7 %) and underweight (35·4 %) among children (below 2 years) in Gadchiroli is like that of the state average, NFHS-5 has found a higher prevalence (30·0 %) of wasting (WHZ < 2 sd) recorded among children below 2 years for Gadchiroli than the state average (25·6 %).

The study identified gender-based disparities in child malnutrition prevalence and the attributing factors be as breast-feeding duration and intra-household food allocation to the differences^(26,27)^. Girls were found to have less dietary diversity^(28)^ and access to food compared with boys, reflecting gender preferences in families^(29)^. Maternal education, poverty and parental attitudes also influence the nutritional status of female children^(26)^.

Government Programs like POSHAN Abhiyan (National Nutrition Mission), Anganwadi Services, Scheme for Adolescent Girls, under Mission Poshan 2.0 and Pradhan Mantri Matru Vandana Yojana under Mission Shakti, aim to address malnutrition issues, especially in tribal areas. Though the Integrated Child Development Services-Common Application Software was created to digitise the records and create a real-time monitoring system for the beneficiaries under the POSHAN Abhiyaan, however, until 2019, only 611 369 Anganwadi workers were equipped with Integrated Child Development Services-Common Application Software^(30)^. Further, the COVID-19 period experienced severe disruption in the transportation and food supply chain, huge shortages and curtailing of employment opportunities, which resulted in worsening the situation of economic crisis among the marginalised and poor population. Thus, the pandemic has resulted in reduced food expenditure and household food insecurity among daily wage earners and farmers^(31,32)^.

Though malnourishment in tribal children showed a declining trend as the prevalence of stunting, wasting and underweight was reduced from 43·8 %, 27·4 % and 45·3 %, respectively, in NFHS-4 to 40·9 %, 23·2 % and 39·5 %, respectively, under NFHS-5, manifold increase in the prevalence of SAM in several Indian districts including in the tribal belts is a public health emergency that requires urgent policy response^(31)^. A study indicated that MUAC is better than WHZ at identifying high-risk children in the community^(33)^. It is also evident that in community-based surveys, both the parameters, i.e. WHZ and MUAC, do not cover the same set of population samples^(34,35)^. Hence, there is a need to develop stand-alone parameters, unlike WHZ or MUAC which ascertain a subset of a population, that can identify all malnourished children^(36)^. The Government of India’s ‘Poshan Tracker’, the largest mobile phone-based nutrition surveillance system in the world, provides transparent data on anthropometric outcomes, the functioning of Anganwadi Centres and the receipt of care services^(37)^. ‘Protocol for Management of Malnourished Children’ has been drafted by MWCD with inputs from MH&FW^(38)^.

Most interventions suggest the treatment of acute malnutrition (SAM) and indicate preventive strategies for chronic malnutrition, as facility-based treatment of acute malnutrition has very limited coverage and incurs more cost^(39)^. At the community level, to manage acute malnutrition, understanding the new guidelines^(40)^ for the therapy of chronic malnutrition and its execution by frontline healthcare providers is necessary. The approach requires many trained staff and a substantial inpatient bed capacity. Besides available facilities, sufficient attention needs to be paid to the quality of care^(41)^.

In India, there is a pressing need for enhanced data collection and monitoring systems to effectively address malnutrition. Additionally, increased investment in infrastructure development is necessary to ensure the timely delivery of vaccines, medicines and hospital facilities^(42)^. The importance of community-level interventions and trained healthcare providers to combat acute malnutrition is also underscored^(43)^. The present study highlights the importance of closer collaboration between various ministries and departments to achieve this goal. The study highlighted gaps in programmatic data for identifying and treating children with SAM among the Indigenous population in India. Further, the present research contributes to understanding the gender gap in child malnutrition in the tribal area. Hence, future studies may address this lacuna in public health research targeting the health and nutritional status of children in tribal populations around the world.

### Strengths and limitations

India’s economy shrank for the first time during the NFHS 5 data collection period due to the nationwide lockdowns from the first wave of the COVID-19 pandemic. This led to disruptions in many nutrition-sensitive programmes, potentially impacting the prevalence of undernutrition. To address this, the current study advocates computing the prevalence of acute malnutrition using both WHZ < 2 sd and MUAC < 12·5 cm parameters to capture all forms of malnourishment. Furthermore, the survey did not collect information on oedema in children under 5 years, which is a limitation of the data.

However, the study focused on the health and nutrition status of children in Anganwadi centres in the selected villages. Household characteristics and dietary information were collected separately, limiting the ability to establish connections between women, children and their household backgrounds. Long-term morbidity or mortality data for children under 5 years were not collected in the survey.

### Conclusions and recommendation

The block-level disparity in the prevalence of malnutrition among children under 5 years in the Gadchiroli district indicates the need for targeted interventions. Monitoring and evaluation of nutrition programs at the block level are crucial to addressing these disparities. Training of health workers to identify and manage malnourished children is essential. Children with critical conditions require specialised nutrition plans and prompt intervention. Besides, AWWs and health workers need to ensure the use of available growth tracker devices and to sensitize the mothers about the health risks associated with malnutrition. Based on the clinical assessment, therapeutic food needs to be provided to the targeted children. Therefore, feeding practices for infants and young children play a crucial role in preventing malnutrition-related illness.

Regular assessment and monitoring of nutrition programs at the block level with data reporting is essential. Qualitative studies can provide valuable insights into nutritional requirements in program areas. Ensuring proper guidelines, followed by frontline health workers and officials, is crucial to address undernutrition effectively. Identifying and treating malnourished children promptly and providing appropriate interventions are key recommendations for improving child nutrition outcomes.

## Supporting information

Patra et al. supplementary material Patra et al. supplementary material

## Footnotes

1 Integrated Child Development Services (ICDS) is one of the most inclusive programmes for addressing child malnutrition and child well-being and development, implemented in 1975 by the Ministry of Women and Child Development, Government of India and supported by the UNICEF [4]. ICDS is operating through Anganwadi Centres (AWCs). An AWC is operated by an Anganwadi Worker (AWW) and an Anganwadi Helper (AWH).

## Supplementary material

For supplementary material accompanying this paper visit https://doi.org/10.1017/S1368980024002465
