# Supplementary material for: Acute malnutrition associated with mid-upper arm circumference among under-five children in tribal areas, India: a cross-sectional study
Source: Public Health Nutr. 2024 Dec 5;28(1):e13. doi: 10.1017/S1368980024002465 (PMC11822597; doi:10.1017/S1368980024002465)

| **Table: Distribution of 0-59 months Children according to Weight for Age, Gadchiroli, Maharashtra, India, 2022** | | | | | | | | |
| --- | --- | --- | --- | --- | --- | --- | --- | --- |
| **Blocks** | **Age group (months)** | **N** | **Severe Underweight** | | **Moderate Underweight** | | **Normal** | |
|  |  |  | **No.** | **%** | **No.** | **%** | **No.** | **%** |
| **Desaiganj** | <12 | 138 | 11 | 8 | 24 | 17.4 | 106 | 76.8 |
|  | 12 to 35 | 266 | 46 | 19.2 | 69 | 28.9 | 151 | 63.2 |
|  | 36 to 59 | 243 | 41 | 16.9 | 82 | 33.7 | 120 | 49.4 |
|  | **0 to 59** | **647** | **98** | **15.1** | **178** | **27.5** | **371** | **57.3** |
| **Bhamragad** | <12 | 83 | 22 | 25.9 | 20 | 23.5 | 43 | 50.6 |
|  | 12 to 35 | 165 | 40 | 24.7 | 44 | 27.2 | 79 | 48.8 |
|  | 36 to 59 | 160 | 37 | 23 | 51 | 31.7 | 72 | 44.7 |
|  | **0 to 59** | **408** | **99** | **24.3** | **115** | **28.2** | **194** | **47.5** |
| ***P values for comparison of nutritional status of 0-59 months children of 2 blocks*** | | | ***15.1% vs 24.3%: P=0.002*** | | ***27.5% vs 28.2%: P=0.805*** | |  | |

**Supplementary Material**

| **Table: Distribution of 0-59 months Children according to Height for Age, Gadchiroli, Maharashtra, India,2022** | | | | | | | | |
| --- | --- | --- | --- | --- | --- | --- | --- | --- |
| **Blocks** | **Age group in months** | **N** | **Severe Stunting** | | **Moderate Stunting** | | **Normal** | |
|  |  |  | **No.** | **%** | **No.** | **%** | **No.** | **%** |
| Desaiganj | <12 | 138 | 17 | 12 | 30 | 21.7 | 91 | 65.9 |
|  | 12 to 35 | 266 | 98 | 41 | 87 | 36.4 | 81 | 33.9 |
|  | 36 to 59 | 243 | 103 | 42 | 48 | 19.8 | 64 | 26.3 |
|  | **0 to 59** | **647** | **218** | **34** | **193** | **29.8** | **236** | **36.5** |
| Bhamragad | <12 | 83 | 24 | 28.2 | 19 | 22.4 | 42 | 49.4 |
|  | 12 to 35 | 165 | 35 | 21.6 | 45 | 27.8 | 82 | 50.6 |
|  | 36 to 59 | 160 | 25 | 15.5 | 49 | 30.4 | 87 | 54 |
|  | **0 to 59** | **408** | **84** | **20.6** | **113** | **27.7** | **211** | **51.7** |
| **P values for comparison of nutritional status of** | | | **Pooled:** | | **Pooled:** | |  | |
| **0-59 months children of** | | | **34.0% vs 20.6%: P=0.001** | | **29.8% vs 27.7%: P=0.464** | |  |  |
| **2 blocks** | | |  | |  | |  |  |

**Annexure**

**1.1 Tape used for measuring MUAC**


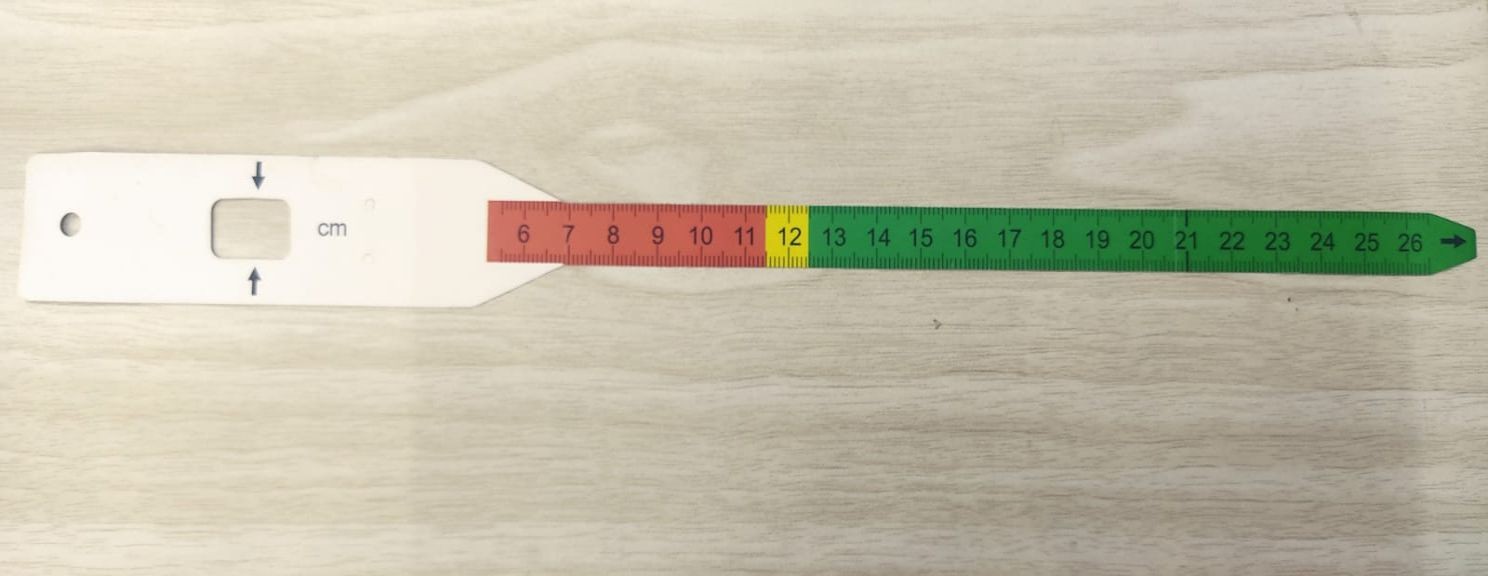

Supplement: Patra et al. supplementary material [file S1368980024002465sup001.docx]
